# The Princess of Wales' Fund for Mrs. Grimwood

**Published:** 1891-07-25

**Authors:** 


### The Princess of Wales' Fund for Mrs. Grimwood.

Her Royal Highness the Princess of Wales cannot fail to be gratified at the proofs that her kindness to the nursing profession is being responded to. Already about 150 nurses have sent in their contributions to the Grimwood Fund, and doubtless this number will be doubled before the Fund is closed. Any nurse wishing to help can send a postal order for 1s. or more to Miss Knollys, at Marlborough House, who acknowledges the following subscriptions: Nurses Blanche, 1s.; E. Schliemann, 1s.; Badder, 1s.; Melinda Smith, 1s.; E. L. White, 1s.; M. A. Plowman, 1s.; Melchied, 2s. 6d.; Foster, 5s.; Sisters Clare Buller, 2s. 6d. Nurses M. E. M., 1s. 6d.; E. Backwith, 2s.; S. Richardson, 2s. 6d.; Isabel Wilson, 2s. 6d.; Jacobs, 1s.; M. J. P., 1s. 6d.; J. Turner, 1s.; A. Underwood, 1s.; Swaby Smith, 1s.; Shaw, 2s.; S. D., 1s.; Edwards, 1s.; A. D., 1s.; M. E. Barber, 1s.; E. M. A. Sherring, 2s. 6d; Walker, 1s.; Groves, 1s.; Assinder, 1s.; Bellamy, 1s.; M. J. W., 1s.; L. Weatherby, 1s.; F. W. W., 1s.; Walters, 1s.; A. J. R., 1s.; A. E. C., 2s. 6d.; Violet, 1s.; Hannah Child, 2s.; Henrietta Walker, 5s. 6d.; M. H. Omerod, Matron, 3s.; E. Stell, 2s.; A. Waller, 1s.; One of the First Thousand, Lincoln Institution, 1s.; Eight Nurses from Seamen's Hospital, Greenwich, 8s.

